# Policy landscape analysis of oral health in Ethiopia

**DOI:** 10.11604/pamj.2026.53.100.49447

**Published:** 2026-02-20

**Authors:** Yilkal Tafere, Achenef Asmamaw Muche, Amare Tariku, Alemnew Athirsaw, Kassahun Alemu

**Affiliations:** 1 Department of Epidemiology and Biostatistics, Institute of Public Health, College of Medicine and Health Sciences, University of Gondar, Gondar, Ethiopia,; 2 Department of Public Health, College of Medicine and Health Sciences, Debre Markos University, Debre Markos, Ethiopia,; 3 Department of Human Nutrition, Institute of Public Health, College of Medicine and Health Sciences, University of Gondar, Gondar, Ethiopia,; 4 Department of Dentistry, Felege Hiwot Comprehensive Specialized Hospital, Bahir Dar, Ethiopia

**Keywords:** Oral diseases, policy, oral health

## Abstract

**Introduction:**

oral health is a crucial yet frequently overlooked component of overall health. While global policies have made progress in reducing oral diseases, many developing countries, including Ethiopia, often do not adequately incorporate oral health into their health policy frameworks. This policy landscape review aimed to synthesize the existing policy documents for oral health in Ethiopia.

**Methods:**

the research employed a mixed-methods design; a desk review of policy documents and key informant interviews. Systematic searching retrieved relevant oral health policies from government websites and the Ministry of Health archives. The desk review used the Policy Triangle Framework for the analysis of the content, context, actors, and processes of currently operational policy documents. While key informant interviews explored challenges affecting oral health policymaking and implementation.

**Results:**

six policy documents were analyzed, and fourteen key informants were interviewed. Ethiopia lacks a comprehensive oral health policy. Oral health was not among the priorities in the health policy. Key gaps identified include: poor integration into broader policies, lack of stakeholder engagement, no dedicated structures in the Ministry of Health, and limited capacity for promotion.

**Conclusion:**

this review highlights the lack of a comprehensive oral health policy and poor integration with general health policies. The development of a national oral health policy could improve its alignment with primary care.

## Introduction

Oral health is a multifaceted aspect of health with functional, physical, and psychosocial elements beyond the mere condition of tooth and gum status [1]. Oral health problems impose a significant burden, causing pain and functional impairment that may threaten people's ability to eat, speak, and socialize [2,3]. If not treated, oral diseases have severe health implications, including infection and life-threatening complications [1]. The global yearly economic cost related to oral diseases and their treatment is estimated at approximately $387 billion, which represents 4.6% of total global health spending. This burden considerably lowers the quality of life due to suffering, limitations in functionality, and societal stigma [1,4,5]. Oral diseases also have a capacity to exacerbate cardiovascular disease and diabetes, contributing to further health and economic burdens [6].

Oral diseases remain a significant public health issue worldwide [7]. Nearly 3.5 billion people globally have oral health conditions, with three-quarters in middle- and low-income nations (LMICs) [7]. Dental cavities alone affect 2.3 billion people in LMICs [8]. Untreated dental caries range from 60% to 80% among children and adults in Ethiopia [9,10]. This disproportionately high oral disease burden in Ethiopia and other LMICs is indicative of huge disparities affecting socio-economically vulnerable groups, i.e., less educated, and rural dwellers [11]. Despite this, access to oral health care and integration into the primary health care system remain major concerns in LMICs [12].

The World Health Organization (WHO) emphasizes the integration of oral health care services into primary health care programs for increased access to the services. The Sustainable Development Goals (SDGs) recognize oral health as a part of the goals of general health, more specifically in SDG 3, that of healthy lives and well-being for all ages [13]. In spite of these worldwide commitments, numerous nations proceed to confront challenges in the integration of oral health into primary health care and Universal Health Coverage systems [14]. The WHO resolution on oral health outlines the high-level policy processes that have led to the prioritization of oral health at the global policy level in the context of health, providing evidence on the dynamics that have pushed oral health onto the international policy agenda [15].

Policy reviews have been inclined to overlook the particular barriers and facilitators to the delivery of oral health services [1,16]. It is crucial to bridge these policy research gaps in order to ensure that oral health is properly prioritized in primary health care and Universal Health Coverage policy development. Ethiopia is striving towards the achievement of the SDGs through its national health policy and Health Sector Development Plans implementation [17,18].

Policy analysis is important in the understanding of oral health system policy operations and to what degree they provide access to oral health care. It identifies gap areas to be employed in reinforcing existing policies and to help develop more effective strategies. Additionally, analysis of policy documents may trigger positive change in oral health governance and provide a framework for achieving universal health coverage. While a comprehensive review of oral health policy content, processes of its development, and their implications on oral health outcomes is lacking today, there is a necessity for the evaluation of the existing policy frameworks for oral health in Ethiopia. Therefore, this study aimed to assess the existing policy frameworks for oral health in Ethiopia and synthesize the extent to which these health policy documents support oral health services.

## Methods

**Study design:** this study employed descriptive qualitative with a desk reviews and key informant interviews approaches throughout data collection and analysis to examine a comprehensive policy landscape analysis of oral health in Ethiopia from January to March 2024. This methodological triangulation enhanced the rigor and credibility of the findings. Recognizing that desk review alone could not capture the complexities of policy implementation and stakeholder engagement, qualitative methods were used to provide context, explore stakeholder perspectives, and clarify the practical realities of oral health. The desk review involved a thorough examination of existing policy documents to assess the inclusion of oral health, gathering relevant data from reliable sources to ensure the analysis is grounded in current literature and reflects the broader context of oral health policy. Key informant interviews provided in-depth insights and contextual details. Including diverse perspectives was crucial for identifying gaps and opportunities in policy implementation, ensuring a thorough analysis of the oral health landscape.

The review utilized Walt and Gilson´s Policy Triangle Framework [19] to analyze the content and integration of oral health policies. This framework was selected for its comprehensive approach to examining the four interconnected elements of health policies: context, content, actors, and processes. The context element investigates the political, economic, social, and cultural factors influencing policy. The content element focuses on the specific components of the policy. The actors element examines stakeholder involvement, while the process element explores how the policy was formulated, communicated, and evaluated over time [19,20].

**Setting:** the study was conducted in Ethiopia, which is an East African country with a diverse population and region-specific health needs. Ethiopia has a decentralized health system, where the Federal Ministry of Health formulates national health policies. The policies are modified and executed by Regional Health Bureaus, which are responsible for addressing region-specific health priorities, overseeing the allocation of resources, and implementing local health interventions. Oral health in Ethiopia is influenced by various factors, including socio-economic conditions, healthcare infrastructure, and public health priorities. The study participants were purposively selected healthcare managers from the Ministry of Health, zonal health departments, district health offices, and facility-level managers, as well as dentists working in various hospitals, and mothers of children with dental caries.

**Policy document search and selection strategy:** health policy documents were accessed from the Ministry of Health's websites. The search strategy aimed to identify all relevant health policy documents available electronically using search terms in both Amharic and English, including “health policy,” “strategic plan,” “guideline,” “oral health care,” “oral health policy,” “Ethiopia,” “dental health,” and “health policy analysis.” The term “health policy documents” refers to any policy statements, strategic plans, or guidelines developed by the Ethiopian Ministry of Health.

Eligibility criteria for the policy documents included: 1) development by the Ethiopian government; 2) recent and currently functional in the public domain; 3) relevant to oral health, either by directly addressing oral health initiatives or by mentioning oral health within the document.

**Key informant interviews:** we employed purposive sampling to select key informants, including dentists and healthcare managers from the Ministry of Health, Zonal Health Departments, District Health Offices, and health facilities to address a broad research question. This method was appropriate as it facilitated the gathering of expert insights and institutional knowledge critical for understanding the complexities of oral health integration. Additionally, we included mothers of schoolchildren diagnosed with dental caries [21] to capture their personal experiences and perspectives on the impact of oral health issues. This inclusion is vital for providing a comprehensive understanding of the challenges faced by families, ensuring that the research addresses both professional insights and the lived experiences of those directly affected.

**Data collection:** data were obtained from policy documents through reviews and key informant interviews. The document review assessed the extent to which oral health problems were highlighted, the comprehensiveness of the content (i.e., the integration of oral health as a core component), the involvement of oral health professionals in policy formulation, and the evaluation mechanisms for oral health. Key informant interviews were conducted in private settings using semi-structured interview guides translated into Amharic. We audio-recorded the interviews on secure, password-protected devices to enable accurate transcription. Interviews continued until data saturation was achieved, at which point no new themes were emerging. The lead interviewer also made field notes throughout the process. Each interview lasts on average 42 minutes.

### Operational definitions

**Well-integrated oral health:** a policy was considered well-integrated when it recognized oral health as a core component of overall health. This included incorporating evidence-based strategies, establishing measurable outcomes, ensuring active stakeholder engagement, and securing sustainable financing. To confirm integration, at least four of these five components need to be met.

**Poorly integrated oral health:** a policy reflected poorly integrated oral health when it failed to acknowledge oral health as a core component. Characteristics of such policies included a lack of evidence-based strategies, the absence of measurable outcomes, minimal stakeholder engagement, and insufficient sustainable financing mechanisms. If at least two of these five components were not met, the policy was deemed poorly integrated.

**Analysis:** the review utilized Walt and Gilson´s Policy Triangle Framework [19] to analyze the content and integration of oral health policies. Data from the policy documents were synthesized narratively, focusing on phrases related to oral health, policy processes, content, context, and gaps in addressing oral health care deficiencies. Our analysis emphasized the integration of oral health as a core component of overall health, incorporating strategies, clear indicators, stakeholder engagement, and sustainable financing.

Key informant interviews were transcribed verbatim from audio recordings into Amharic and then translated into English by the principal investigator and with other qualitative research experts from Debre Markos University to see data consistency. Transcription began during data collection, with the majority completed shortly after fieldwork. The finding was returned to a few (2) participants. Data were analyzed using ATLAS.ti version 9, where each transcript was carefully screened and coded. These codes were subsequently grouped into categories, subthemes, and themes. Findings from interviews with different stakeholders were triangulated with the results of desk reviews.

**Ethical consideration:** the Institutional Review Board of the University of Gondar, College of Medicine and Health Sciences, and Specialized Hospital ethically approved this study with reference number (R/T/T/C/Eng./151/18/2023) Gondar, Ethiopia. Additionally, researchers contacted the participants, explained the objectives of the study, and obtained written informed consent. The conventional ethical considerations for conducting research (preserving anonymity and ensuring confidentiality) were adhered.

## Results

**Figure 1:**
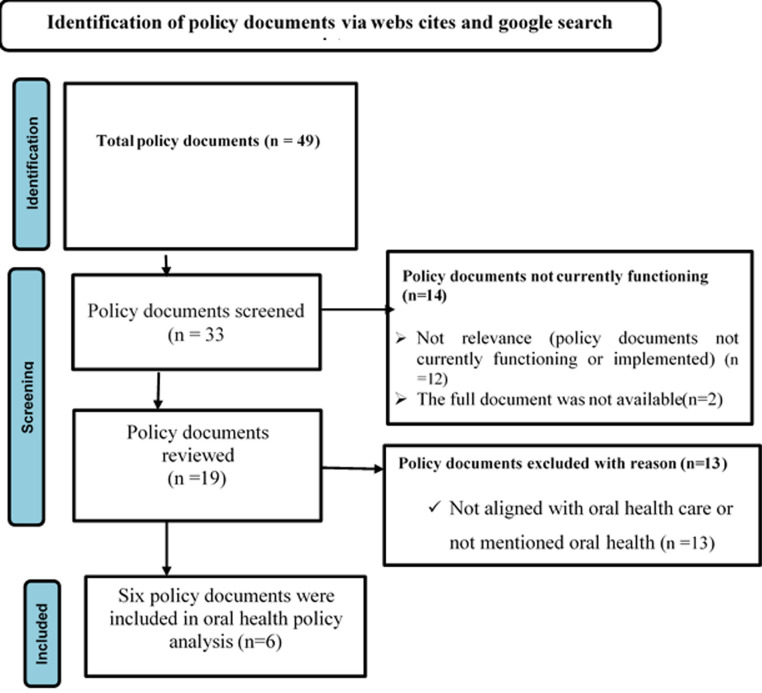
flow diagram for the selection of policy documents for oral health policy analysis in Ethiopia, 2024

**Descriptive analysis:** the search yielded 49 entries. After removing duplicates and screening according to the inclusion criteria, six policy documents were included for full-text review and analysis. A flow chart of the search and selection process is presented in Figure 1. Of the available health policy documents, six [18,22-26] mentioned oral health. These documents were further analyzed to assess the status of oral health elements, the degree to which risk factors for oral health were addressed, the prioritization of oral health within the health system, and the availability of strategies for integrating oral health care into other health policy agendas. The extent to which oral health was outlined varied among the documents, as evidenced by the statements extracted from them. A total of 14 key informants were interviewed, of whom four (28.6%) were female (Table 1).

**Table 1:** characteristics of participants in key informant interviews on oral health policy in Ethiopia, 2024 (n=14)

Variables		Numbers
Sex	Male	10
	Female	4
Age in years	20-29	2
	30-39	8
	≥40	4
Occupation	Dentist	4
	Healthcare manager	6
	Teacher	1
	Farmer	2
	Merchant	1
Residence	Rural	2
	Urban	12

**Oral health policy findings:** this review observed that a comprehensive stand-alone policy document for preventing and controlling oral diseases in Ethiopia is lacking [17]. The qualitative results indicated that oral health was not a priority in the national health agenda. A healthcare manager from the Ministry of Health remarked, *“oral health is not regarded as a fundamental element of universal health coverage; it often gets overlooked for more pressing health issues”*. Managers at the facility level expressed similar thoughts, pointing out *“oral health is frequently neglected in resource allocation and implementation.”*

The Ministry of Health and related government offices have not shown strong leadership in advancing the oral health agenda, leading to a lack of prioritization. The reviewed documents [18,22-26] demonstrate that the Ministry is responsible for setting up guidelines, developing guidelines, preparing action plans, and coordinating national health services; however, there is currently no dedicated oral health structure within the Ministry, which contributes to significant coordination gaps in oral health services. Leadership remains a crucial role, as pointed out by the participants at all levels. A national level health care manager expressed, *“there is no oral health structure in the Ministry of Health like other health problems, which is due to lack of attention by the leadership, which created major coordination gaps in oral health services*.” Managers also noted the shortage of dentists as a key system-level problem.

### Oral health policy analysis using the Walt and Gilson policy triangle

**Context:** key contextual factors identified include socio-economic conditions, insufficient human resources for health, and regional variations in health indicators. These determinants are driven by the need to address maternal and child mortality, the burden of communicable and non-communicable diseases, and malnutrition. The policy documents align with Ethiopia´s global commitments and macro-economic development framework, yet critical factors related to oral health remain unaddressed (Table 2).

**Table 2:** document review using the policy triangle framework: context, process, content, and stakeholders in the formulation of health policy documents in Ethiopia, 2024

Policy document	Context	Process	Content	Stakeholders
National strategic action plan for the prevention and control of major non-communicable diseases	Lack of integration of oral health into NCD strategies; insufficient data on the burden of oral diseases	Situational analysis, consultative meetings, and workshops	No specific policy actions identified to address oral health; no directives for integrating oral health; no specific indicators for oral health; no monitoring strategies for oral health	WHO, UNICEF, UNDP, Ministry of Health, regional health bureaus, academic institutions, and community representatives. Involvement of oral health professionals does not indicate
Health Sector Transformation Plan (HSTP-II)	Insufficient focus on oral health; burden of oral diseases not clearly indicated	Consultative workshops, technical working groups, field visits, and situational analysis.	Oral health not prioritized; no specific indicators for oral health; no actions identified to address oral health; no monitoring strategies for oral health expressed	The World Bank, WHO, Ministry of Health, regional health bureaus, and community members. Oral health professionals are not included in the development process
School health program framework	Lack of government priority regarding oral health; burden of oral diseases acknowledged	Situational analysis, discussions with experts, and brief school visits	No indicators for oral health; no specific guidelines or packages for oral health; no monitoring strategies for oral health	Ministry of Health, Ministry of Education, regional health bureaus, and program managers. Oral health professionals are not specifically mentioned
Essential Health Services Package (EHSP)	The burden of oral health problems is not clearly stated	Prioritization process based on national health policy; consultation with experts	No specific EHSP components referring to oral health; no indicators to assess oral health; no monitoring strategies for oral health expressed	WHO, Ministry of Health, regional health bureaus, universities, and community representatives. Involvement of oral health professionals is not mentioned
National WASH and environmental health strategy	Lack of emphasis on oral hygiene within WASH strategies; insufficient integration of oral health	Situational analysis, consultations, workshops, and expert discussions	Lack of strategies for oral health implementation; lack of indicators to measure oral health; WASH initiatives do not adequately promote oral health; no monitoring strategies for oral health are expressed	WHO, USAID, UNICEF, Water Aid Ethiopia, Ministry of Health, regional health bureaus, sector ministries, and academic institutions. The contribution of oral health professionals is not indicated
National Antenatal Care (ANC) guideline	Insufficient integration of oral health during ANC; burden of oral diseases not clearly indicated	Situational analysis, consultative meetings with experts, and workshops	No specific core packages of ANC referring to oral health care; no specific policy actions identified for oral health; no intention to monitor oral health care expressed	WHO, UNICEF, Ministry of Health, regional health bureaus, gynecologists, obstetricians, midwives, pediatricians, public health specialists, and academic institutions. Oral health professionals are not included in the working group

NCD: non-communicable diseases; WASH: water, sanitation, and hygiene; WHO: World Health Organization; UNICEF: United Nations Children's Fund; UNDP: United Nations Development Programme

The analysis of current health policy documents [18,22-26] did not provide the nation's oral health situation, disease burden, and dental healthcare-seeking habits. There is a lack of recent epidemiological data about oral health. The key informant interview with the dentist provided critical insights into the burden of oral health problems in Ethiopia and the need for situational analysis. He pointed out that the country still faces a high prevalence of preventable oral diseases such as dental caries, periodontal diseases, and oral cancers, which often progress to advanced and debilitating stages because of limited access to preventive and curative services. *“I saw so many patients presenting with advanced tooth decay, gum infections, and even late-stage oral cancers that could have been easily managed if caught early."*

He further added that the absence of reliable data on the epidemiology of oral diseases and related risk factors is a big challenge in designing evidence-informed strategies to solve the problem. *“Without insight into the true magnitude of an oral health burden, its inequities across demographic groups, and the social determinants, designing evidence-based policies and programs remains challenging to address this public health issue.”* One significant bottleneck that prevents policymakers and health authorities from allocating resources, prioritizing high-impact interventions, and tracking progress over time is the absence of a comprehensive situational analysis.

**Process:** the policy development process included situational analysis, consultative forums, workshops, technical working groups, and field visits. Stakeholder involvement in the development of key health policy documents in Ethiopia is crucial in ensuring that the policies are comprehensive, contextually relevant, and address the various health needs of the population in an efficient manner. Key stakeholders like the WHO, UNICEF, UNDP, the Ministry of Health, the Ministry of Education, regional health bureaus, and civil society organizations played significant roles in the development of policies for health in Ethiopia. These stakeholders contributed through technical and financial support. However, in the reviewed documents [18,22-26], the policy documents did not reflect the active involvement of key stakeholders, including oral health professionals and situational analysis with respect to oral diseases (Table 2).

Oral health professionals were excluded from most health policy documents development processes, which is supposed to create an opportunity for the oral health workforce to influence the policy process. The policy documents do not demonstrate a systematic, evidence-based approach to identifying and addressing oral health priorities. In the reviewed documents, the oversight and coordination of oral health activities across different programs and stakeholders are limited. The result of the key informant interview with a dentist underlined the critical importance of national oral health policy. A participant stated, *“we do not have a clear, overarching policy framework to guide oral healthcare planning and implementation. As a result, interventions have been rolled out in a fragmented way."*As shown by the dentists, the problem was worsened by a lack of resource allocation, public awareness, and integration into general healthcare.

**Content:** health policy documents like the National Strategic Action Plan for the Prevention and Control of Major Non-Communicable Diseases [22], Health Sector Transformation Plan II [18], School Health Program Framework [25], Essential Health Services Package of Ethiopia [23], National WASH and Environmental Health Strategy [26], and National Antenatal Care Guideline [24], mentioned oral health. Nonetheless, these policy documents have no specific oral health care implementation plans (Table 2).

This study indicates that the nation currently lacks sufficient, all-inclusive oral health targets and indicators to properly address oral health needs and track improvements. Most of the policy documents reviewed were related to maternal and child health, non-communicable diseases, nutrition-related issues, and infectious diseases such as pneumonia, diarrhea, malaria, and tuberculosis, among others. The reviewed policy documents set general health targets without clearly defining the specific oral health outcomes they aim to achieve, such as reducing the prevalence of dental caries, preventing periodontal (gum) diseases, and increasing access to basic dental services. Quantifiable oral health indicators that would allow progress to be tracked and evaluated are absent from the strategies [18,22-26].

The Essential Health Services Package document better by incorporating some coverage of oral health services; this document lacked quantifiable objectives and performance indicators to track progress in advancing population-level oral health. Other policy frameworks, such as the National Strategic Action Plan for Non-Communicable Diseases and the Health Sector Transformation Plan II, only briefly and without outlining specific, measurable targets or detailed initiatives. The School Health Program, National WASH and Environmental Health Strategy, and National Antenatal Care Guideline demonstrated a more limited focus on oral health considerations, though they did not entirely omit the topic. Those policy documents lack strategic objectives and detailed indicators to evaluate.

Participants of the qualitative interview pointed out the poor integration of oral healthcare services within the broader primary care system; participants explained that, *“too many Ethiopians simply don't have the means or ability to see a dentist regularly. This leads to preventable problems escalating into a major or debilitating stage.”* Participant recognized the importance of oral health to overall patient wellbeing. As one dentist stated, *“oral health is absolutely critical. It affects nutrition, chronic conditions, and quality of life. We can't treat the whole patient without addressing their oral health needs*.” Mothers also reported that their children's dental caries had a significant impact on their well-being. A mother said: *“the toothaches were just awful for my son. He cries in pain, and he cannot eat or sleep properly.”* Participants noted impacts on their children's self-esteem due to the appearance of decayed teeth.

Most of the mothers had a relatively low oral hygiene knowledge and self-efficacy about good oral hygiene practices for their children. Their knowledge regarding recommended brushing frequency and techniques for brushing children's teeth was low. One mother states that *“I do not think that I am really sure if I am brushing my child's teeth properly. I just kind of wipe their teeth with a toothbrush, but I do not know if I'm doing it thoroughly enough."*

The current Ethiopian Health Insurance Scheme did not cover most of the oral health care services, such as dentures, crowns, bridges, implants, and root canal treatments (except those required due to infections), which are not covered by the insurance scheme [17]. Key informant interviews with dentists showed that major barriers preventing patients from having access to oral health care services included the lack of dental insurance and high costs for treatment. As one dentist commented, *“the reality is that dental care is still viewed as a luxury, not a necessity. And until we fix the fundamental problems with access and affordability, we'll continue to see patients delay or limit their treatment-seeking behavior."*

The barriers to preventive oral care identified by mothers in the in-depth interviews were a lack of dental insurance, costs, and difficulty accessing dentists. Mothers of children stated their considerable lack of economic access to oral health care services. A mother explained, *“we have health insurance, but it doesn't cover much dental work. I have to pay hundreds of Ethiopian Birr (local currency) just to get my children´s cavities filled.”* In addition to this, many mothers discussed the emotional distress they experienced watching their children suffer from dental pain. A participant shared, *“it's heartbreaking to see your child in pain and not be able to afford the care they need."*

## Discussion

Over the past three decades, Ethiopia has developed better policies and strategies related to health challenges within the country. Oral health, at present times, is considered the main determinant of health, well-being, and quality of life, and therefore fundamental to universal health coverage. Yet, our findings register some key gaps: notably, the lack of a stand-alone oral health policy; the lack of integration of oral health services into broader health policies; the lack of stakeholder engagement; inadequate financing for oral health; and the lack of an oral health unit within the Ministry of Health.

This study identified a serious gap in oral health policy in Ethiopia. Such as the absence of an independent policy on oral health, which has had serious implications for the country in terms of addressing the increasing burden of oral diseases. Without a dedicated policy, the prioritization, coordination, and implementation of targeted oral health programs have been hindered. This finding is reinforced by the results of the key informant interview of the current study. Participant described that the lack of a clear strategic direction has become piecemeal and ad hoc rather than demonstrating a systemic approach toward improving oral health. Oral health issues have often been conquered by other pressing public health concerns, such as the existence of high maternal and child mortality, high burden of communicable diseases, and institutional challenges, poor governance within the healthcare system, i.e., lack of an oral health unit within the Ministry of Health.

These gaps have hindered the political will and resources allocation for developing policies to address oral health as a distinct priority and integrate it effectively into the broader health plan, and drive the necessary policy and programmatic interventions [4,27-29]. Unlike Ethiopia, other African countries, Malawi [30] and Nigeria [31] had a standalone oral health policy. The possible reason in those countries where oral health is a priority in their policy documents might be attributed to various factors, including stronger political will, a higher burden of oral diseases, and active advocacy groups that champion oral health. These factors likely contribute to a greater recognition of oral health as a critical component of overall health. Standalone oral health policies could increase access to quality oral healthcare services, especially for underserved populations. Consequently, it would lead to a better prevention and management of oral diseases contribute to improved health outcomes. Oral health policies also promote oral health and targeted prevention programs; hence, they empower the communities.

Another weakness highlighted in the process of developing policy documents is the insufficiency of evidence-based analysis regarding the oral health situation in Ethiopia. It would appear that the non-availability of comprehensive data on the oral health of the population and the healthcare system is impeding the policy formulation process. However, WHO suggests that the formulation of evidence-based oral health policies should be guided by an in-depth analysis of trends in prevalence, severity, and distribution of oral diseases, alongside reviewing social, economic, environmental, and behavioral determinants for oral health in the country [15]. In the absence of an in-depth understanding of the context-specific oral health challenges, including their underpinning drivers and resources and capacities available, the provisions of these policies might not be representative of the real situation on the ground. The current study's interview results support this finding. Based on their personal experience, participants highlighted the enormous burden of oral health problems in the country and underscored the need for comprehensive situational analyses to inform policy decisions at all levels.

The analysis indicated that oral health was poorly integrated into the broader healthcare. In the process of developing health policies, a country neglected oral health to make an integral part of such policy documents [18,22-26]. Results also indicate that mothers have low knowledge about oral hygiene for their children. However, it is now widely recognized that integrating oral health into the broader primary healthcare is vital to improve oral health services [1,7,32]. This has the potential to undermine synergies within the health system if oral health is fragmented, leading to a lack of clear linkages, coordination, and resource allocation to ensure the effective delivery of integrated oral health services. It could also further contribute to a lack of oral health literacy among caregivers, which stands out as one of the most important barriers to ensuring good oral health outcomes for children. Proper integration of oral health into the primary health care improves access to preventive education and services that can take advantage of the existing infrastructures of the health care to promote oral health awareness and stimulate the adoption of healthy behaviors by mothers for the oral health of children from an early age.

On the same note, the World Health Organization document addresses a policy development process for oral health that incorporates several stakeholders, such as oral health professionals. However, the review of health policy documents underscores a critical gap in the meaningful engagement of oral health professionals [33], in the integration and development of oral health policies and strategies. Despite oral health professionals being at the forefront of delivering essential services and having invaluable, context-specific insights, the analysis found limited evidence of their participation in the policymaking process. This finding is supported by the key informant interview results of the current study, which showed that there was no engagement of oral professionals in policy formulation. This has led to policies that lack proper incorporation of realities, needs, and views of the people who will implement the interventions and care for the population.

One of the key challenges identified in this policy analysis is the structural gap of oral health within the Ministry of Health to district level. This structural gap has several implications for the coordination of oral health programs. Participants in the key informant interviews highlighted that without a designated unit for oral health, it become challenging to ensure that oral healthcare service receives adequate attention, resource allocation, and integration into the broader healthcare. The review also revealed that the lack of an oral health unit has contributed to the fragmentation and suboptimal coordination of oral health initiatives across different programs. The centralized oral health unit is crucial to provide strategic direction to ensure the implementation of oral health policies and interventions. This finding is similar with finding in India and South Africa [34,35], which did not have a dedicated oral health division within the Ministry of Health.

The strengths of this review are triangulation of data sources, through documents reviewed and by key informant interviews, to enhance the credibility and reliability of the findings. This encapsulates contextual insight obtained from interviews with diverse stakeholders. The limitations include the scope of the document review being limited to publicly available government sources.

## Conclusion

The analysis of the health policy landscape has revealed critical gaps in the country's approach to oral health, including the complete absence of a standalone oral health policy. Oral health integration within broader health policies is fragmented. Additionally, mothers had limited knowledge about proper oral hygiene practices for their children and were unaware of the recommended frequency for brushing their children's teeth. The policy analysis also identified the absence of an institutional oral health unit within the Ministry of Health. Ethiopia needs to establish a dedicated oral health policy with a coherent strategy and roadmap. Efforts are required to improve mothers´ knowledge regarding their children's oral care. Establishing a dedicated oral health unit is critical for mainstreaming oral health into Ethiopia´s primary healthcare system.

### What is known about this topic

- Oral health is closely linked to the overall health of the population; poor oral health can lead to serious health issues, including cardiovascular diseases, diabetes, and respiratory infections;
- In Ethiopia, oral health care providers are concentrated in urban areas; oral health disparities exist among different socio-economic groups.

### What this study adds

- The study highlights the urgent need for a comprehensive oral health policy or strategies tailored to the country-specific situation;
- This study highlights the critical importance of establishing oral health units within the Ministry of Health with adequate budgets at all administrative levels.
